# 3-Ethyl­sulfinyl-2-(3-fluoro­phen­yl)-5-phenyl-1-benzo­furan

**DOI:** 10.1107/S1600536813015924

**Published:** 2013-06-12

**Authors:** Hong Dae Choi, Pil Ja Seo, Uk Lee

**Affiliations:** a Department of Chemistry, Dongeui University, San 24 Kaya-dong, Busanjin-gu, Busan 614-714, Republic of Korea; b Department of Chemistry, Pukyong National University, 599-1 Daeyeon 3-dong, Nam-gu, Busan 608-737, Republic of Korea

## Abstract

In the title compound, C_22_H_17_FO_2_S, the dihedral angles between the mean plane [r.m.s. deviation = 0.005 (1) Å] of the benzo­furan ring system and the pendant 3-fluoro­phenyl and phenyl rings are 23.92 (5) and 32.44 (5)°, respectively. In the crystal, mol­ecules are linked by two weak C—H⋯O(sulfin­yl) hydrogen bonds and a C—H⋯π inter­action, forming a sheet, which lies in the *ab* plane. A π–π inter­action between the benzene and furan rings of neighbouring mol­ecules [centroid–centroid distance = 3.976 (2) Å] links the mol­ecules into inversion dimers and connects adjacent sheets, resulting in a three-dimensional network.

## Related literature

For background information and the crystal structures of related compounds, see: Choi *et al.* (2006 ▶, 2010 ▶).
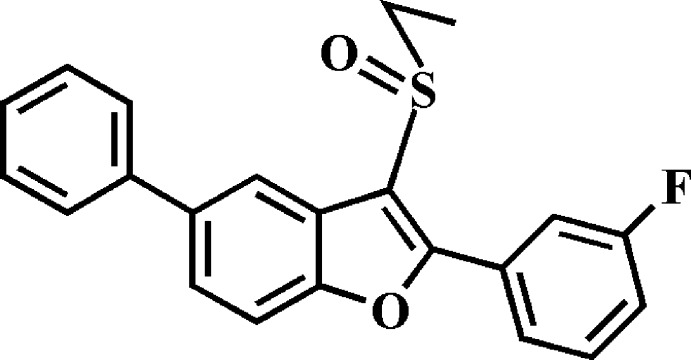


## Experimental

### 

#### Crystal data

C_22_H_17_FO_2_S
*M* *_r_* = 364.42
Monoclinic, 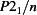

*a* = 12.6447 (3) Å
*b* = 7.1680 (2) Å
*c* = 19.2382 (5) Å
β = 100.592 (2)°
*V* = 1713.99 (8) Å^3^
*Z* = 4
Mo *K*α radiation
μ = 0.21 mm^−1^
*T* = 173 K
0.38 × 0.25 × 0.18 mm

#### Data collection

Bruker SMART APEXII CCD diffractometer
Absorption correction: multi-scan (*SADABS*; Bruker, 2009 ▶) *T*_min_ = 0.684, *T*_max_ = 0.746
16390 measured reflections
4269 independent reflections
3406 reflections with *I* > 2σ(*I*)
*R*_int_ = 0.037

#### Refinement

*R*[*F* ^2^ > 2σ(*F* ^2^)] = 0.046
*wR*(*F* ^2^) = 0.128
*S* = 1.04
4269 reflections
236 parameters
H-atom parameters constrained
Δρ_max_ = 0.85 e Å^−3^
Δρ_min_ = −0.41 e Å^−3^

### 

Data collection: *APEX2* (Bruker, 2009 ▶); cell refinement: *SAINT* (Bruker, 2009 ▶); data reduction: *SAINT*; program(s) used to solve structure: *SHELXS97* (Sheldrick, 2008 ▶); program(s) used to refine structure: *SHELXL97* (Sheldrick, 2008 ▶); molecular graphics: *ORTEP-3* (Farrugia, 2012 ▶) and *DIAMOND* (Brandenburg, 1998 ▶); software used to prepare material for publication: *SHELXL97*.

## Supplementary Material

Crystal structure: contains datablock(s) global, I. DOI: 10.1107/S1600536813015924/go2092sup1.cif

Structure factors: contains datablock(s) I. DOI: 10.1107/S1600536813015924/go2092Isup2.hkl

Supplementary material file. DOI: 10.1107/S1600536813015924/go2092Isup3.cml

Additional supplementary materials:  crystallographic information; 3D view; checkCIF report

## Figures and Tables

**Table 1:** Hydrogen-bond geometry (Å, °) *Cg*1 is the centroid of the C9–C14 phenyl ring.

*D*—H⋯*A*	*D*—H	H⋯*A*	*D*⋯*A*	*D*—H⋯*A*
C12—H12⋯O2^i^	0.95	2.49	3.166 (3)	128
C21—H21*A*⋯O2^ii^	0.99	2.58	3.367 (3)	136
C14—H14⋯*Cg*1^iii^	0.95	2.66	3.466 (3)	143

Symmetry codes: (i) 
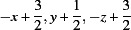
; (ii) 
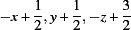
; (iii) 
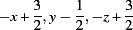
.

**Fig. 1.**
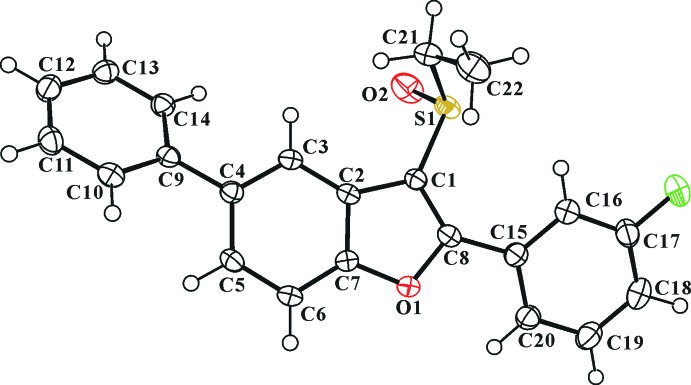
The molecular structure of the title compound with the atom numbering scheme. Displacement ellipsoids are drawn at the 50% probability level. H atoms are presented as small spheres of arbitrary radius.

**Fig. 2.**
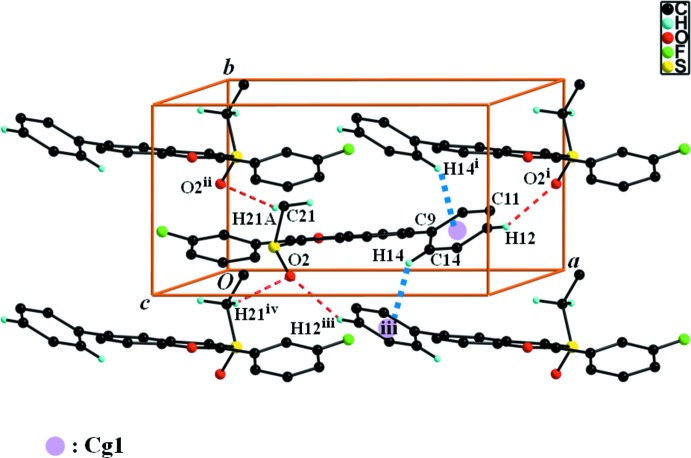
A view of the C—H···O and C—H···π interactions (dotted lines) in the crystal structure of the title compound. H atoms non-participating in hydrogen-bonding were omitted for clarity. [Symmetry codes: (i) - x + 3/2, y + 1/2, - z + 3/2; (ii) - x + 1/2, y + 1/2, - z + 3/2; (iii) - x + 3/2, y -1/2, - z + 3/2; (iv) - x + 1/2, y - 1/2, - z + 3/2.]

**Fig. 3.**
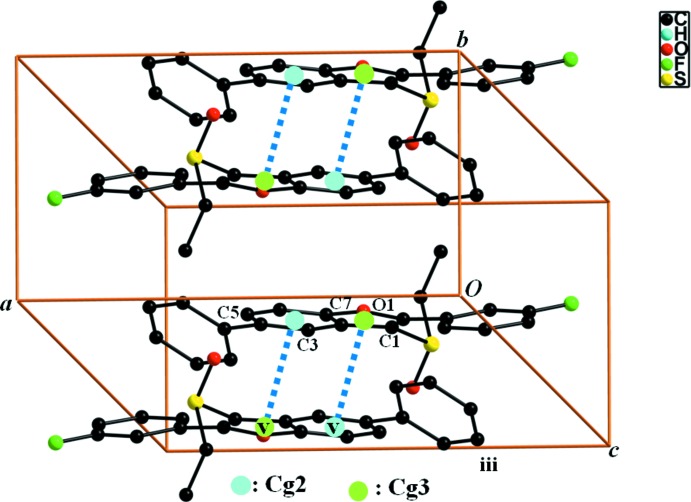
A view of the π–π interactions (dotted lines) in the crystal structure of the title compound. All H atoms were omitted for clarity. [Symmetry codes: (v) - x + 1, - y, - z + 1.]

## supplementary crystallographic information

### Comment

As a part of our ongoing study of 5-phenyl-1-benzofuran derivatives containing 2-methyl-3-methylsulfinyl (Choi *et al.*, 2006) and [2-(4-fluorophenyl)-3-methylsulfinyl] (Choi *et al.*, 2010) substituents, we report herein the crystal structure of the title compound.

In the title molecule (Fig. 1), the benzofuran unit is essentially planar, with a mean deviation of 0.005 (1) Å from the least-squares plane defined by the nine constituent atoms. The dihedral angles between the mean plane of the benzofuran ring system and the pendant 3-fluorophenyl and phenyl rings are 23.92 (5) and 32.44 (5)°, respectively. In the crystal structure (Fig. 2), molecules are connected by the C12—H12···O2^i^ [symmetry code: (i) -x+3/2,y+1/2,-z+3/2] weak hydrogen bond and the C14—H14···Cg1^iii^ [symmetry code: (iii) -x+3/2,y-1/2,-z+3/2], C—H···π interactions (Table 1), (Cg1 is the centroid of the C9–C14 phenyl ring). This links the molecules into a chain of glide related molecules which runs parallel to the *b*-axis. The chains are linked to form a two dimensional sheet by the C21–H21A···O2^ii^ [symmetry code: (ii) -x+1/2,y+1/2,-z+3/2] weak hydrogen bond (Table 1). This sheet lies in the *ab-plane*. In the crystal packing (Fig. 3), a π–π interaction between the benzene and furan rings of neighbouring molecules into inversion dimers, with a Cg2···Cg3^v^ [Symmetry code: (v) -x+1,-y,-z+1]distance of 3.976 (2) Å and interplanar distance of 3.515 (2) Å resulting in a slippage of 1.858 (2) Å (Cg2 and Cg3 are the centroids of the C2-C7 benzene ring and the C1/C2/C7/O1/C8 furan ring, respectively), links adjacent sheets into a three-dimensional network.

### Experimental

3-Chloroperoxybenzoic acid (77%, 202 mg, 0.9 mmol) was added in small portions to a stirred solution of 3-ethylsulfanyl-2-(3-fluorophenyl)-5-phenyl-1-benzofuran (278 mg, 0.8 mmol) in dichloromethane (30 mL) at 273 K. After being stirred at room temperature for 5h, the mixture was washed with saturated sodium bicarbonate solution and the organic layer was separated, dried over magnesium sulfate, filtered and concentrated at reduced pressure. The residue was purified by column chromatography (hexane–ethyl acetate, 2:1 v/v) to afford the title compound as a colorless solid [yield 62%, m.p. 445–446 K; *R*f = 0.51 (hexane–ethyl acetate, 2:1 v/v)]. Single crystals suitable for X-ray diffraction were prepared by slow evaporation of a solution of the title compound in acetone at room temperature.

### Refinement

All H atoms were positioned geometrically and refined using a riding model, with C–H = 0.95 Å for aryl, 0.99 Å for methylene, 0.98Å for methyl H atoms. *U*_iso_(H) = 1.2*U*_eq_(C) for aryl and methylene H atoms, and 1.5*U*_eq_(C) for methyl H atoms. The positions of methyl hydrogens were optimized rotationally.

### Crystal data

**Table:**

C_22_H_17_FO_2_S	*F*(000) = 760
*M**_r_* = 364.42	*D*_x_ = 1.412 Mg m^−^^3^
Monoclinic, *P*2_1_/*n*	Melting point: 445 K
Hall symbol: -P 2yn	Mo *K*α radiation, λ = 0.71073 Å
*a* = 12.6447 (3) Å	Cell parameters from 4233 reflections
*b* = 7.1680 (2) Å	θ = 2.2–28.0°
*c* = 19.2382 (5) Å	µ = 0.21 mm^−^^1^
β = 100.592 (2)°	*T* = 173 K
*V* = 1713.99 (8) Å^3^	Block, colourless
*Z* = 4	0.38 × 0.25 × 0.18 mm

### Data collection

**Table:**

Bruker SMART APEXII CCD diffractometer	4269 independent reflections
Radiation source: rotating anode	3406 reflections with *I* > 2σ(*I*)
Graphite multilayer monochromator	*R*_int_ = 0.037
Detector resolution: 10.0 pixels mm^-1^	θ_max_ = 28.3°, θ_min_ = 1.8°
φ and ω scans	*h* = −16→16
Absorption correction: multi-scan (*SADABS*; Bruker, 2009)	*k* = −9→9
*T*_min_ = 0.684, *T*_max_ = 0.746	*l* = −25→25
16390 measured reflections	

### Refinement

**Table:**

Refinement on *F*^2^	Primary atom site location: structure-invariant direct methods
Least-squares matrix: full	Secondary atom site location: difference Fourier map
*R*[*F*^2^ > 2σ(*F*^2^)] = 0.046	Hydrogen site location: difference Fourier map
*wR*(*F*^2^) = 0.128	H-atom parameters constrained
*S* = 1.04	*w* = 1/[σ^2^(*F*_o_^2^) + (0.0567*P*)^2^ + 1.1638*P*] where *P* = (*F*_o_^2^ + 2*F*_c_^2^)/3
4269 reflections	(Δ/σ)_max_ < 0.001
236 parameters	Δρ_max_ = 0.85 e Å^−^^3^
0 restraints	Δρ_min_ = −0.41 e Å^−^^3^

### Special details

**Table:**

Geometry. All esds (except the esd in the dihedral angle between two l.s. planes) are estimated using the full covariance matrix. The cell esds are taken into account individually in the estimation of esds in distances, angles and torsion angles; correlations between esds in cell parameters are only used when they are defined by crystal symmetry. An approximate (isotropic) treatment of cell esds is used for estimating esds involving l.s. planes.
Refinement. Refinement of F^2^ against ALL reflections. The weighted R-factor wR and goodness of fit S are based on F^2^, conventional R-factors R are based on F, with F set to zero for negative F^2^. The threshold expression of F^2^ > 2sigma(F^2^) is used only for calculating R-factors(gt) etc. and is not relevant to the choice of reflections for refinement. R-factors based on F^2^ are statistically about twice as large as those based on F, and R- factors based on ALL data will be even larger.

### Fractional atomic coordinates and isotropic or equivalent isotropic displacement parameters (Å^2^)

**Table:**

	*x*	*y*	*z*	*U*_iso_*/*U*_eq_	
S1	0.28388 (4)	0.20679 (7)	0.65076 (2)	0.02778 (14)	
F1	−0.08545 (10)	0.2673 (2)	0.49260 (7)	0.0538 (4)	
O1	0.37450 (10)	0.22928 (19)	0.46484 (6)	0.0258 (3)	
O2	0.34178 (13)	0.0589 (2)	0.69642 (8)	0.0450 (4)	
C1	0.34858 (14)	0.2327 (2)	0.57766 (9)	0.0218 (3)	
C2	0.46328 (14)	0.2461 (2)	0.57892 (9)	0.0216 (3)	
C3	0.55575 (14)	0.2587 (2)	0.63076 (9)	0.0224 (3)	
H3	0.5509	0.2585	0.6795	0.027*	
C4	0.65569 (14)	0.2718 (2)	0.61010 (9)	0.0221 (3)	
C5	0.66128 (15)	0.2675 (3)	0.53742 (9)	0.0267 (4)	
H5	0.7297	0.2752	0.5239	0.032*	
C6	0.57064 (15)	0.2525 (3)	0.48550 (9)	0.0277 (4)	
H6	0.5751	0.2484	0.4368	0.033*	
C7	0.47340 (14)	0.2437 (2)	0.50788 (9)	0.0231 (4)	
C8	0.29968 (14)	0.2219 (2)	0.50834 (9)	0.0234 (4)	
C9	0.75598 (14)	0.2881 (2)	0.66393 (9)	0.0221 (3)	
C10	0.84465 (15)	0.3880 (3)	0.64995 (9)	0.0265 (4)	
H10	0.8411	0.4457	0.6051	0.032*	
C11	0.93742 (15)	0.4040 (3)	0.70038 (10)	0.0301 (4)	
H11	0.9968	0.4728	0.6899	0.036*	
C12	0.94449 (16)	0.3206 (3)	0.76596 (10)	0.0304 (4)	
H12	1.0082	0.3325	0.8006	0.037*	
C13	0.85745 (16)	0.2192 (3)	0.78057 (10)	0.0278 (4)	
H13	0.8617	0.1605	0.8253	0.033*	
C14	0.76453 (15)	0.2037 (3)	0.73008 (9)	0.0249 (4)	
H14	0.7055	0.1342	0.7407	0.030*	
C15	0.18887 (15)	0.1985 (2)	0.47093 (9)	0.0243 (4)	
C16	0.10093 (16)	0.2510 (3)	0.50082 (10)	0.0309 (4)	
H16	0.1107	0.3089	0.5460	0.037*	
C17	−0.00028 (16)	0.2166 (3)	0.46299 (11)	0.0339 (4)	
C18	−0.01985 (16)	0.1354 (3)	0.39756 (11)	0.0353 (4)	
H18	−0.0913	0.1122	0.3736	0.042*	
C19	0.06749 (16)	0.0883 (3)	0.36737 (10)	0.0341 (4)	
H19	0.0563	0.0333	0.3217	0.041*	
C20	0.17108 (15)	0.1205 (3)	0.40320 (9)	0.0284 (4)	
H20	0.2306	0.0895	0.3816	0.034*	
C21	0.32241 (17)	0.4276 (3)	0.69310 (10)	0.0336 (4)	
H21A	0.3068	0.4261	0.7417	0.040*	
H21B	0.4008	0.4462	0.6965	0.040*	
C22	0.26270 (18)	0.5865 (3)	0.65229 (12)	0.0397 (5)	
H22A	0.2734	0.5820	0.6031	0.060*	
H22B	0.2900	0.7052	0.6737	0.060*	
H22C	0.1858	0.5758	0.6535	0.060*	

### Atomic displacement parameters (Å^2^)

**Table:**

	*U*^11^	*U*^22^	*U*^33^	*U*^12^	*U*^13^	*U*^23^
S1	0.0303 (3)	0.0299 (2)	0.0262 (2)	0.00221 (19)	0.01334 (18)	0.00413 (18)
F1	0.0266 (7)	0.0891 (12)	0.0475 (8)	0.0094 (7)	0.0112 (6)	−0.0037 (7)
O1	0.0228 (6)	0.0364 (7)	0.0189 (6)	−0.0008 (5)	0.0053 (5)	−0.0013 (5)
O2	0.0521 (10)	0.0453 (9)	0.0417 (8)	0.0148 (8)	0.0195 (7)	0.0176 (7)
C1	0.0214 (8)	0.0244 (8)	0.0203 (7)	0.0006 (7)	0.0061 (6)	0.0004 (6)
C2	0.0231 (9)	0.0229 (8)	0.0202 (7)	0.0005 (7)	0.0079 (6)	−0.0005 (6)
C3	0.0255 (9)	0.0243 (8)	0.0183 (7)	0.0006 (7)	0.0065 (6)	−0.0003 (6)
C4	0.0233 (9)	0.0220 (8)	0.0216 (8)	0.0017 (7)	0.0052 (7)	0.0002 (6)
C5	0.0235 (9)	0.0345 (10)	0.0242 (8)	0.0002 (7)	0.0101 (7)	0.0018 (7)
C6	0.0273 (9)	0.0387 (10)	0.0187 (8)	−0.0007 (8)	0.0088 (7)	−0.0005 (7)
C7	0.0231 (9)	0.0273 (9)	0.0188 (8)	−0.0001 (7)	0.0039 (6)	−0.0008 (6)
C8	0.0243 (9)	0.0238 (8)	0.0235 (8)	0.0016 (7)	0.0082 (7)	−0.0002 (6)
C9	0.0235 (8)	0.0217 (8)	0.0220 (8)	0.0012 (7)	0.0067 (6)	−0.0022 (6)
C10	0.0283 (9)	0.0270 (9)	0.0256 (8)	−0.0001 (7)	0.0091 (7)	0.0016 (7)
C11	0.0250 (9)	0.0299 (9)	0.0365 (10)	−0.0043 (8)	0.0085 (8)	−0.0018 (8)
C12	0.0260 (9)	0.0324 (10)	0.0310 (9)	0.0007 (8)	0.0002 (7)	−0.0042 (8)
C13	0.0302 (10)	0.0284 (9)	0.0243 (8)	0.0018 (8)	0.0034 (7)	0.0009 (7)
C14	0.0255 (9)	0.0255 (9)	0.0243 (8)	−0.0015 (7)	0.0058 (7)	−0.0002 (7)
C15	0.0253 (9)	0.0227 (8)	0.0245 (8)	0.0010 (7)	0.0038 (7)	0.0018 (7)
C16	0.0269 (10)	0.0389 (11)	0.0268 (9)	0.0027 (8)	0.0043 (7)	−0.0007 (8)
C17	0.0244 (9)	0.0425 (11)	0.0356 (10)	0.0052 (8)	0.0076 (8)	0.0047 (9)
C18	0.0269 (10)	0.0356 (11)	0.0393 (10)	−0.0013 (8)	−0.0044 (8)	0.0015 (9)
C19	0.0343 (11)	0.0340 (10)	0.0305 (9)	0.0032 (8)	−0.0033 (8)	−0.0045 (8)
C20	0.0291 (10)	0.0288 (9)	0.0266 (8)	0.0036 (8)	0.0033 (7)	−0.0026 (7)
C21	0.0339 (11)	0.0419 (11)	0.0260 (9)	0.0044 (9)	0.0083 (8)	−0.0070 (8)
C22	0.0455 (13)	0.0325 (11)	0.0445 (12)	0.0029 (9)	0.0172 (10)	−0.0007 (9)

### Geometric parameters (Å, º)

**Table:**

S1—O2	1.4817 (15)	C11—C12	1.384 (3)
S1—C1	1.7620 (17)	C11—H11	0.9500
S1—C21	1.805 (2)	C12—C13	1.390 (3)
F1—C17	1.357 (2)	C12—H12	0.9500
O1—C7	1.371 (2)	C13—C14	1.385 (3)
O1—C8	1.375 (2)	C13—H13	0.9500
C1—C8	1.365 (2)	C14—H14	0.9500
C1—C2	1.449 (2)	C15—C16	1.394 (3)
C2—C3	1.393 (2)	C15—C20	1.397 (2)
C2—C7	1.396 (2)	C16—C17	1.373 (3)
C3—C4	1.396 (2)	C16—H16	0.9500
C3—H3	0.9500	C17—C18	1.368 (3)
C4—C5	1.413 (2)	C18—C19	1.381 (3)
C4—C9	1.487 (2)	C18—H18	0.9500
C5—C6	1.379 (3)	C19—C20	1.383 (3)
C5—H5	0.9500	C19—H19	0.9500
C6—C7	1.377 (2)	C20—H20	0.9500
C6—H6	0.9500	C21—C22	1.506 (3)
C8—C15	1.462 (2)	C21—H21A	0.9900
C9—C14	1.395 (2)	C21—H21B	0.9900
C9—C10	1.397 (2)	C22—H22A	0.9800
C10—C11	1.383 (3)	C22—H22B	0.9800
C10—H10	0.9500	C22—H22C	0.9800
			
O2—S1—C1	107.30 (8)	C11—C12—H12	120.4
O2—S1—C21	107.26 (10)	C13—C12—H12	120.4
C1—S1—C21	98.12 (9)	C14—C13—C12	120.11 (17)
C7—O1—C8	106.78 (13)	C14—C13—H13	119.9
C8—C1—C2	107.00 (15)	C12—C13—H13	119.9
C8—C1—S1	125.46 (14)	C13—C14—C9	121.22 (17)
C2—C1—S1	127.14 (13)	C13—C14—H14	119.4
C3—C2—C7	119.06 (16)	C9—C14—H14	119.4
C3—C2—C1	136.21 (15)	C16—C15—C20	119.25 (17)
C7—C2—C1	104.74 (15)	C16—C15—C8	122.05 (16)
C2—C3—C4	119.01 (15)	C20—C15—C8	118.70 (16)
C2—C3—H3	120.5	C17—C16—C15	117.98 (18)
C4—C3—H3	120.5	C17—C16—H16	121.0
C3—C4—C5	119.53 (16)	C15—C16—H16	121.0
C3—C4—C9	120.51 (15)	F1—C17—C18	118.49 (18)
C5—C4—C9	119.96 (16)	F1—C17—C16	117.62 (19)
C6—C5—C4	122.15 (17)	C18—C17—C16	123.88 (19)
C6—C5—H5	118.9	C17—C18—C19	117.90 (18)
C4—C5—H5	118.9	C17—C18—H18	121.1
C7—C6—C5	116.63 (16)	C19—C18—H18	121.1
C7—C6—H6	121.7	C18—C19—C20	120.46 (18)
C5—C6—H6	121.7	C18—C19—H19	119.8
O1—C7—C6	125.62 (15)	C20—C19—H19	119.8
O1—C7—C2	110.77 (15)	C19—C20—C15	120.47 (18)
C6—C7—C2	123.60 (16)	C19—C20—H20	119.8
C1—C8—O1	110.71 (15)	C15—C20—H20	119.8
C1—C8—C15	135.10 (16)	C22—C21—S1	111.11 (14)
O1—C8—C15	114.15 (14)	C22—C21—H21A	109.4
C14—C9—C10	117.87 (16)	S1—C21—H21A	109.4
C14—C9—C4	120.94 (16)	C22—C21—H21B	109.4
C10—C9—C4	121.19 (15)	S1—C21—H21B	109.4
C11—C10—C9	120.98 (16)	H21A—C21—H21B	108.0
C11—C10—H10	119.5	C21—C22—H22A	109.5
C9—C10—H10	119.5	C21—C22—H22B	109.5
C10—C11—C12	120.53 (17)	H22A—C22—H22B	109.5
C10—C11—H11	119.7	C21—C22—H22C	109.5
C12—C11—H11	119.7	H22A—C22—H22C	109.5
C11—C12—C13	119.29 (18)	H22B—C22—H22C	109.5
			
O2—S1—C1—C8	125.48 (17)	C3—C4—C9—C14	31.9 (2)
C21—S1—C1—C8	−123.52 (17)	C5—C4—C9—C14	−147.46 (18)
O2—S1—C1—C2	−46.25 (18)	C3—C4—C9—C10	−148.25 (17)
C21—S1—C1—C2	64.74 (17)	C5—C4—C9—C10	32.4 (2)
C8—C1—C2—C3	−179.2 (2)	C14—C9—C10—C11	−0.6 (3)
S1—C1—C2—C3	−6.2 (3)	C4—C9—C10—C11	179.48 (16)
C8—C1—C2—C7	0.57 (19)	C9—C10—C11—C12	0.2 (3)
S1—C1—C2—C7	173.54 (14)	C10—C11—C12—C13	0.4 (3)
C7—C2—C3—C4	1.0 (2)	C11—C12—C13—C14	−0.6 (3)
C1—C2—C3—C4	−179.22 (19)	C12—C13—C14—C9	0.1 (3)
C2—C3—C4—C5	−1.5 (3)	C10—C9—C14—C13	0.5 (3)
C2—C3—C4—C9	179.17 (15)	C4—C9—C14—C13	−179.62 (16)
C3—C4—C5—C6	0.6 (3)	C1—C8—C15—C16	25.1 (3)
C9—C4—C5—C6	179.99 (17)	O1—C8—C15—C16	−157.38 (17)
C4—C5—C6—C7	0.6 (3)	C1—C8—C15—C20	−154.7 (2)
C8—O1—C7—C6	179.11 (18)	O1—C8—C15—C20	22.8 (2)
C8—O1—C7—C2	−0.12 (19)	C20—C15—C16—C17	2.5 (3)
C5—C6—C7—O1	179.75 (17)	C8—C15—C16—C17	−177.30 (18)
C5—C6—C7—C2	−1.1 (3)	C15—C16—C17—F1	179.68 (18)
C3—C2—C7—O1	179.53 (15)	C15—C16—C17—C18	−0.6 (3)
C1—C2—C7—O1	−0.28 (19)	F1—C17—C18—C19	178.58 (19)
C3—C2—C7—C6	0.3 (3)	C16—C17—C18—C19	−1.1 (3)
C1—C2—C7—C6	−179.53 (18)	C17—C18—C19—C20	0.9 (3)
C2—C1—C8—O1	−0.7 (2)	C18—C19—C20—C15	1.0 (3)
S1—C1—C8—O1	−173.79 (12)	C16—C15—C20—C19	−2.8 (3)
C2—C1—C8—C15	176.92 (19)	C8—C15—C20—C19	177.05 (17)
S1—C1—C8—C15	3.8 (3)	O2—S1—C21—C22	−176.95 (14)
C7—O1—C8—C1	0.5 (2)	C1—S1—C21—C22	72.02 (15)
C7—O1—C8—C15	−177.63 (14)		

### Hydrogen-bond geometry (Å, º)

Cg1 is the centroid of the C9–C14 phenyl ring.

**Table:**

*D*—H···*A*	*D*—H	H···*A*	*D*···*A*	*D*—H···*A*
C12—H12···O2^i^	0.95	2.49	3.166 (3)	128
C21—H21*A*···O2^ii^	0.99	2.58	3.367 (3)	136
C14—H14···*Cg*1^iii^	0.95	2.66	3.466 (3)	143

Symmetry codes: (i) −*x*+3/2, *y*+1/2, −*z*+3/2; (ii) −*x*+1/2, *y*+1/2, −*z*+3/2; (iii) −*x*+3/2, *y*−1/2, −*z*+3/2.
